# Factors affecting feeding choices in infants and toddlers in northern Jordan: A cross-sectional study

**DOI:** 10.1371/journal.pone.0347995

**Published:** 2026-04-24

**Authors:** Wajdi Amayreh, Mohammad Al-Magableh, Jomana Alsulaiman, Mahdi Alshboul, Maan Amayreh, Ahmad Al-Maqableh, Razan Qasem, Tamara Al-Nemrat

**Affiliations:** 1 Department of Pediatrics, Family Medicine and Obstetrics & Gynecology, Faculty of Medicine, Yarmouk University, Irbid, Jordan; 2 Acute Medical Unit, Epsom General Hospital, Epsom, United Kingdom; 3 Jordan Royal Medical Services, Amman, Jordan; 4 Faculty of Medicine, Yarmouk University, Irbid, Jordan; 5 Ministry of Health, Hashemite Kingdom of Jordan, Irbid, Jordan; Menzies School of Health Research: Charles Darwin University, AUSTRALIA

## Abstract

**Background:**

Breastfeeding is a key determinant of infant health and survival; however, exclusive breastfeeding (EBF) rates remain low worldwide. Various maternal, infant, and socioeconomic factors influence the feeding practices.

**Objective:**

The main objective of this study was to identify maternal, infant, and socioeconomic determinants of infant feeding practices during the first six months of life among mothers in northern Jordan.

**Methods:**

A prospective cross-sectional study was conducted at Princess Rahma and Prince Rashid Hospitals in Irbid City, northern Jordan, from December 2023 to February 2024. Mothers of healthy infants aged 6–24 months participated in a survey that gathered information on their demographics, feeding practices, and other infant-related details. Statistical analyses were performed to identify the associations and key predictors of feeding type.

**Results:**

Among the 508 mothers who participated in this study, 29.9% were exclusively breastfeeding, 46.5% used mixed feeding, and 23.6% opted for formula feeding. The key factors influencing these choices include maternal health issues, work hours, and infant birth weight. Maternal illness was identified as the strongest predictor of exclusive artificial feeding (AOR = 12.72; 95% CI: 4.10–39.45; P < 0.001). Low birth weight (<2.5 kg) was also associated with higher odds of artificial feeding (AOR = 4.75; 95% CI: 2.08–10.88; p < 0.001). Maternal employment significantly increased the likelihood of mixed feeding compared with EBF (AOR = 3.54; 95% CI: 1.65–7.60; p = 0.001). Surprisingly, no significant correlation was found between maternal education, family income, cultural factors, and feeding methods.

**Conclusion:**

This study highlighted the low exclusive breastfeeding rate, emphasizing the need for improved support systems to encourage breastfeeding in the form of workplace accommodations and healthcare counseling to address barriers to its practice.

## Introduction

According to the World Health Organization (WHO), breastfeeding is one of the most effective ways to ensure an infant’s health and survival. However, despite the WHO’s recommendations for exclusive breastfeeding (EBF) during the first six months of life, global rates, as shown in Table 1, remain suboptimal [1]. In low- and middle-income countries, only 37% of infants meet this standard [2].

**Table 1:** Rates of exclusive breastfeeding in infants 0-5 months old according to UNICEF (2017).

Country’s Name	Rates of exclusive breastfeeding according to UNICEF (2017)
**Afghanistan**	43%
**Pakistan**	37%
**Turkey**	30%
**Malaysia**	28%
**Somalia**	5%
**Djibouti**	3%

In Jordan, while 34% of infants begin breastfeeding within the first hour after birth, the duration of exclusive breastfeeding is far from ideal. Only 24% of newborns aged 2–3 months continue to be exclusively breastfed [3].

Although previous studies have explored individual factors influencing breastfeeding practices in Jordan, there is still a lack of comprehensive data on the combined impact of maternal working hours, health status, and socioeconomic factors on feeding decisions in northern Jordan, particularly in Irbid, which is the second most populous governorate in the country [4].

Breast milk is considered the ideal food for newborn infants because of its ability to meet their complete nutritional needs. To highlight some of the benefits of breastfeeding, it is associated with improved overall infant health and immune development, fewer incidences of gastrointestinal diseases, and lower mortality rates than formula-fed infants [5,6]. In addition to supplying essential nutrients to growing infants, breast milk also serves as a source of commensal bacteria, which further boosts infant health by preventing pathogen adhesion and promoting the colonization of beneficial microbes in the gut. In addition, it has an increased bioavailability of minerals, vitamins, and proteins [5,6].

Infant feeding modes play a significant role in the overall health and development of infants [7]. These modes are influenced by several factors, including maternal education, cultural background, and socioeconomic status [8].

Many mothers may change their feeding preference from breastfeeding to formula feeding for several reasons or perceived obstacles, such as a lack of social support, difficulties in balancing breastfeeding with work commitments, physical discomfort, concerns about changes in body appearance, and worries about postpartum weight retention [9]. Therefore, it is essential to understand how various factors influence mothers’ choices regarding infant feeding.

Infant feeding methods include breastfeeding and formula feeding, each with its own advantages and challenges. Breastfeeding is known for its health benefits for both mothers and children, as it provides infants with essential nutrients, antibodies, immune cells, and bioactive components [5]. Furthermore, research has shown that breastfed infants experience fewer ear infections, respiratory issues, and stomach problems than non-breastfed infants [6]. In addition to its nutritional and immune benefits, it strengthens the bond and emotional connection between the mother and child through “skin-to-skin” contact and oxytocin release [10]. This hormonal release further supports faster postpartum recovery by promoting uterine contractions and helping the uterus return to its pre-pregnancy size [11]. Additionally, mothers who breastfed their babies weighed, on average, 8 kg less than those who did not breastfeed six years later. Breastfeeding is also associated with a general reduction in infant illness and hospitalization rates [12]. However, the most fascinating benefit of breastfeeding is the decreased risk of various maternal cancers. Extending breastfeeding by 12 months is linked to a 4.3% decrease in the incidence of invasive breast cancer. If breastfeeding becomes nearly universal, it could prevent 20,000 breast cancer-related deaths annually. Moreover, prolonged breastfeeding is associated with a reduction in the risk of ovarian cancer, ranging from 18% to 30% [2]. In contrast, formula milk does not offer some of the immunological and nutritional benefits of breast milk. [13]

The influence of maternal education, culture, and other factors cannot be overlooked, as they play an important role in determining feeding choices. Educational background and cultural traditions can impact a mother’s decision to breastfeed or formula feed [14]. Similarly, socioeconomic status is another key determinant, as it can affect whether an infant has a supportive environment for breastfeeding [15]. Therefore, understanding these dynamics is essential for developing effective breastfeeding promotion strategies.

This study aimed to understand how factors such as culture, education, and socioeconomic status influence infant feeding preferences among mothers in northern Jordan. We also explored the relationships between these factors and overall infant health across different feeding modes and identified the key determinants and barriers to exclusive breastfeeding (EBF).

## Methods

### Study design

This was a prospective, observational, cross-sectional survey conducted during the study period from the 1st of December 2023 to the 25^th^ of February 2024.

### Study setting

The study was conducted in the outpatient pediatric clinics of Princess Rahma and Prince Rashid Military Hospital, two of the largest tertiary public hospitals in northern Jordan.

### Study population and sample

The target population comprised mothers who were the primary caregivers of infants aged 6–24 months and who attended outpatient pediatric clinics during the study period.

The inclusion criteria comprised Jordanian mothers aged 18 years or older with healthy singleton infants aged 6–24 months who attended the clinics during the study period and were willing to participate. Additionally, the mothers needed to be able to read and understand Arabic, the language of the survey. Infants with major congenital malformations or severe chronic conditions, such as cleft palates or metabolic diseases requiring special diets that could affect feeding practices, were excluded from the study.

A minimum sample size of 475 was calculated using Cochran’s formula with a 95% confidence interval, 4.5% margin of error, and a conservatively estimated population proportion of 50%.

### Study questionnaire and data collection

A structured, electronic, self-administered questionnaire developed from the literature and adapted to the local context was used in this study. The survey consisted of two sections that required no more than 15 minutes for the mothers to complete. The first section gathered sociodemographic data, including maternal age, education, occupation, working hours, accommodation, and monthly family income. The second section collected information on maternal, infant, and feeding practices, such as birth weight, chosen feeding method during the first six months of life (categorized as exclusive breastfeeding, mixed feeding, or exclusive artificial feeding), duration of exclusive breastfeeding, weaning details, any maternal illnesses or medications that prevented breastfeeding, and whether mothers received medical counseling on breastfeeding.

In this study, artificial feeding referred to the use of commercial infant formulas. Mixed feeding refers to the use of formula milk alongside breast milk in the first six months of life.

The research team consisted of trained medical students who visited the study hospitals as permitted. During their visits, they approached all available mothers, introduced themselves, assessed their eligibility, and offered them the opportunity to participate in the survey. Mothers who agreed to participate were provided with additional details. Verbal and written consent were obtained from all participants. The medical students were responsible for data collection and asked the mothers to complete the electronic self-administered questionnaire on the students’ phones or tablets. They were also available to clarify any unclear questions in the survey. Each questionnaire took approximately 15 minutes to complete. A total of 517 mothers agreed to participate in the survey, with 311 responses gathered from Princess Rahma Pediatric Hospital and 206 from the Prince Rashid Military Hospital. After excluding nine responses due to missing information, 508 questionnaire responses were included in the final data analysis.

### Data analysis

Statistical analysis of the 508 responses was performed using SPSS 29 and Jamovi 2.7 software. Descriptive statistics were calculated for the participants’ sociodemographic characteristics and feeding outcomes. Chi-square tests were used to assess the associations between participant characteristics and the feeding method of choice during the first 6 months of life (exclusive breastfeeding, exclusive artificial feeding, and mixed feeding). To identify predictors of infant feeding practices, variables with a p-value <0.20 in univariable analyses, as well as variables deemed relevant based on the literature review (maternal age and educational level), were fitted into a multinomial logistic regression model with feeding type as the dependent variable (reference category: exclusive breastfeeding) and reported adjusted odds ratios (AORs) with 95% confidence intervals (CIs). All variables were treated as categorical variables. Statistical significance was set at P ≤ 0.05. The model fit for the logistic regression was evaluated using deviance, Akaike Information Criterion (AIC), Bayesian Information Criterion (BIC), and pseudo-R^2^ measures (Nagelkerke).

### Ethical approval

The study protocol was approved by the Institutional Review Board (IRB) at Yarmouk University in Jordan on the 30^th^ of November 2023 (approval number: IRB/2023/623), and each participant provided verbal and written informed consent.

## Results

### Participant characteristics

A total of 508 responses from the study hospitals were included in the data analysis, with 304 (59.8%) responses from Princess Rahma Hospital and 204 (40.2%) from the Prince Rashid Military Hospital.

Most mothers (59.6%) were aged 25–35 years, while 24.0% were over 35 years, and 16.3% were younger than 25 years. A considerable proportion of the mothers (39.6%) held a bachelor’s degree, while 39.7% had completed year 12 (Tawjihi in Jordan), 18.1% had an educational level below year 12, and 2.6% had a postgraduate degree. Most fathers worked in the private sector (48.2%), followed by the military (30.1%) and public (19.1%), and (2.6%) were unemployed. A large proportion (80.5%) of mothers were unemployed, while 15.6% worked 1–8 h and 3.9% worked more than 8 h. In total, 19.5% of the mothers worked long hours outside their homes.

The study sample consisted of 38.6% female and 61.4% male infants. Most infants (81.9%) weighed between 2.5 and 4 kg at birth, with 15% weighing less than 2.5 kg and 3.1% weighing > 4 kg. A notable proportion (39.8%) of infants were products of preterm pregnancies, whereas the majority (60.2%) were products of full-term pregnancies.

A substantial portion of families (64.6%) had a monthly income of less than 400 Jordanian Dinars (JD), with 21.5% earning 400–700, 10.6% earning 700–1000 JD, and only 3.3% earning more than 1000 JD. The sociodemographic characteristics of the study sample, presented in Table 2, provide a comprehensive overview of various variables related to the mother, child, and family.

**Table 2:** Sociodemographic characteristics of the study sample (N = 508).

Variable	Categories	Overall n%
**Mother’s age**	<25	83 (16.3%)
	25-35	303 (59.6%)
	>35	122 (24.0%)
**Child’s age**	6-11 months	101 (19.9%)
	12-17 months	236 (46.5%)
	18-24 months	171 (33.7%)
**Child’s Gender**	Female	196 (38.6%)
	Male	312 (61.4%)
**Mother’s Educational Level**	Less than year 12	92 (18.1%)
	Year 12	202(39.7%)
	Bachelor’s degree	201 (39.6%)
	Postgraduate	13 (2.6%)
**Father’s Job**	Unemployed	13 (2.6%)
	Military sector	153 (30.1%)
	Private sector	245 (48.2%)
	Public sector	97 (19.1%)
**Mother’s working hours outside the house**	Unemployed	409 (80.5%)
	1-8 hrs/day	79 (15.6%)
	>8 hrs/day	20 (3.9%)
**Living Arrangement**	Separate household	389 (76.6%)
	Shared household with parents/in-laws	119 (23.4%)
**Monthly Income (JDs)**	<400	328 (64.6%)
	400-700	109 (21.5%)
	700-1000	54 (10.6%)
	>1000	17 (3.3%)
**Preterm or Full-term Pregnancy**	Preterm Pregnancy	202 (39.8%)
	Full-term Pregnancy	306 (60.2%)
**Weight Categories at birth**	<2.5 Kg	76 (15%)
	2.5-4 Kg	416 (81.9%)
	> 4 Kg	16 (3.1%)

### Infant feeding practices during the first year of life

Infant feeding practices during the first year of life are presented in Table 3. Mixed feeding (Formula and Breastfeeding) was the most common feeding method in the first 6 months of life (46.5%), followed by exclusive breastfeeding (29.9%) and exclusive artificial feeding (23.6%). Among infants who received breast milk (N = 388) (76.4%), 58.5% were breastfed for six months or less, 21.0% continued breastfeeding for 7–12 months, and 20.5% were breastfed for > 12 months. The most commonly reported reason for using artificial milk was the need to use formula to support breastfeeding (63.8%), followed by maternal-related factors (29.5%), and 6.7% of mothers reported no specific reason. Regarding weaning, 42.2% of infants were introduced to solid foods before six months of age, 53.6% were weaned between six and 12 months, and 4.2% after 12 months.

**Table 3:** Patterns of infant feeding during the first year of life (N = 508).

Variables	Categories	Overall (n%)
**Feeding method during the first 6 months of life (N = 508).**	Exclusive Artificial feeding	120 (23.6%)
	Exclusive Breastfeeding	152 (29.9%)
	Mixed feeding	236 (46.5%)
**Breastfeeding duration among infants who received breast milk (N = 388)**	0-6 months	227 (58.5%)
	7-12 months	81(21.0%)
	More than 12 months	80 (20.5%)
**Reasons for Artificial (Formula) Feeding (N = 356)**	Use of formula to support breastfeeding	227 (63.8%)
	Maternal-related factors	105 (29.5%)
	No specific reason reported	24 (6.7%)
**Daily frequency of milk feeding (N = 508)**	3-6	266 (52.4%)
	7-10	194 (38.2%)
	More than 10	48 (9.4%)
**Age at introduction of solid foods (N = 508)**	<6 months	214 (42.2%)
	6–12 months	272 (53.6%)
	>12 months	22 (4.2%)

### Maternal and infant health-related characteristics

Table 4 summarizes maternal and child health and other related variables. Most mothers (87.8%) reported no maternal illness or medication that would contraindicate breastfeeding, whereas 12.2% reported such conditions. Less than half of the mothers (46.1%) received healthcare counseling on breastfeeding, while 53.9% did not receive any medical counseling. Most infants (96.4%) visited hospitals and clinics or were admitted between one and five times. A small percentage, 2.4%, attended six to ten visits, while only 1.2% of infants had more than ten visits. The primary causes of these hospital visits varied, with respiratory causes accounting for 145 (28.5%), gastrointestinal causes for 38 (7.5%), and other causes for 325 (64%) cases.

**Table 4:** Maternal and infant health, breastfeeding counseling, and other characteristics of the study sample (N = 508).

Variable	Categories	Overall n%
**Maternal illness or medication contraindicating breastfeeding.**	No	446 (87.8%)
	Yes	62 (12.2%)
**Received healthcare counseling on breastfeeding.**	No	274 (53.9%)
	Yes	234 (46.1%)
**Number of healthcare visits during the first 12 months of life (outpatient/inpatient).**	1-5	490 (96.4%)
	6-10	12 (2.4%)
	More than 10	6 (1.2%)
**Primary cause of healthcare visits**	GI causes	38 (7.5%)
	Respiratory causes	145 (28.5%)
	Others	325 (64%)

### Associations between feeding type and sociodemographic characteristics

Table 5 explores the associations between feeding type during the first 6 months of life and demographic variables in the study population, employing the chi-square test with a significance level of 0.05. No statistically significant association was found between maternal age and feeding method during the first 6 months of life (p = 0.291). Maternal education level was not significantly associated with the feeding method during the first 6 months (p = 0.359). A statistically significant association was found between mothers’ working hours outside the home and their feeding methods during the first 6 months (p = 0.019). No significant association was found between monthly income and the nutritional method during the first 6 months (p = 0.067).

**Table 5:** Feeding type within 6 months and possible correlation with the study demographic variables using the chi-square test (N = 508).

		Method of nutrition used during the first 6 months				
Variable	Categories	Artificial feeding	Breastfeeding	Mixed	Test value	p-value (Chi-Square Test)
**Mother’s age**	**<25**	18 (15.0%)	26 (17.1%)	39 (16.5%)	4.965	0.291
	**25-35**	81 (67.5%)	89 (58.6%)	133 (56.4%)		
	**>35**	21(17.5%)	37(24.3%)	64(27.1%)		
**Mother’s Educational Level**	**Bachelor’s degree**	55 (45.8%)	59 (38.8%)	87 (36.9%)	6.602	0.359
	**Less than Year 12**	21 (17.5%)	29 (19.1%)	42 (17.8%)		
	**Postgraduate**	3 (2.5%)	1 (0.7%)	9 (3.8%)		
	**Year 12**	41 (34.2%)	63 (41.4%)	98 (41.5%)		
**Mother’s working hours outside the house**	**unemployed**	99 (82.5%)	134 (88.2%)	176 (74.6%)	**11.806**	**0.019**
	**1-8**	18 (15.0%)	14 (9.2%)	47 (19.9%)		
	**>8**	3 (2.5%)	4 (2.6%)	13 (5.5%)		
**Living Arrangement**	**Separate household**	95 (79.2%)	112 (73.7%)	182 (77.1%)	1.196	0.550
	**Shared household with parents/in-laws**	25 (20.8%)	40 (26.3%)	54 (22.9%)		
**Monthly Income**	**<400**	77 (64.2%)	97 (63.8%)	154 (65.3%)	11.772	0.067
	**400-700**	20 (16.7%)	43 (28.3%)	46 (19.5%)		
	**700-1000**	18 (15.0%)	9 (5.9%)	27 (11.4%)		
	**>1000**	5(4.2%)	3(2.0%)	9(3.8%)		
**Weight Categories when born**	<2.5 Kg	36 (30.3%)	11 (7.5%)	29 (12.3%)	31.2	0.000
	2.5-4 Kg	79 (65.5%)	137 (89.8%)	200 (84.7%)		
	> 4 Kg	5 (4.2%)	4 (2.7%)	7 (3.0%)		
**Preterm or Full-term Pregnancy**	Preterm Pregnancy	56 (46.7%)	52 (34.2%)	94 (39.8%)	4.345	0.114
	Full-term Pregnancy	64 (53.3%)	100 (65.8%)	142 (60.2%)		

A significant association was found between infants’ birth weight categories and the method of nutrition during the first 6 months (p = 0.000). Infants weighing less than 2.5 kg at birth were more likely to be artificially fed, whereas those weighing 2.5–4 kg or > 4 kg were more likely to be breastfed. The chi-square test also revealed no significant association between pregnancy duration (preterm or full-term) and the method of nutrition during the first 6 months (p = 0.114).

### Associations between feeding type and health-related variables

Table 6 illustrates the potential correlation between feeding types within the first 6 months and various suggested medical variables. A highly significant association was found between maternal illness or medication contraindicating breastfeeding and the method of nutrition during the first 6 months (p = 0.000). A statistically significant association was observed between receiving medical counseling on breastfeeding and the method of nutrition used during the first 6 months (p = 0.049). On the other hand, there was no significant association between the number of hospital visits and the method of nutrition during the first 6 months (p = 0.090). However, we observed that artificially fed infants tended to have more hospital visits, particularly 1–5 visits. No significant association was found between the cause of hospital visits and the method of nutrition during the first 6 months (p = 0.16).

**Table 6:** Feeding type within 6 months and possible correlation with suggested medical variables using chi-square test (N = 508).

		Method of nutrition used during the first 6 months				
Variable	Categories	Artificial feeding	Breastfeeding	Mixed	Test value	p-value (Chi-Square Test)
**Maternal illness or medication contraindicating breastfeeding.**	**No**	87 (72.5%)	148 (97.4%)	211 (89.4%)	**39.772**	**0.000**
	**Yes**	33 (27.5%)	4 (2.6%)	25 (10.6%)		
**Received healthcare counseling on breastfeeding.**	**No**	53 (44.2%)	86 (56.6%)	135 (57.2%)	**6.05**	**0.049**
	**Yes**	67 (55.8%)	66 (43.4%)	101 (42.8%)		
**Number of healthcare visits during the first 12 months of life (outpatient/inpatient).**	**1-5**	111 (92.4%)	147 (96.6%)	232 (98.3%)	8.054	0.090
	**6-10**	6 (5.0%)	3 (2.0%)	3 (1.3%)		
	**More than 10**	3 (2.5%)	2 (1.4%)	1 (0.4%)		
**Primary cause of healthcare visits**	**GI causes**	6 (5.0%)	8 (5.4%)	24 (10.2%)	6.59	0.16
	**Respiratory causes**	40 (32.8%)	39 (26.5%)	66 (28.0%)		
	**Others**	74 (62.2%)	105 (68.1%)	146 (61.8%)		

### Independent predictors of feeding type during the first six months

A multinomial logistic regression was performed to identify the major barriers and predictors of infant feeding methods during the first six months of life. Exclusive Breastfeeding (EBF) served as the reference category to compare the odds of exclusive artificial feeding and mixed feeding. The overall model was statistically significant compared to the null model (X^2^(40) = 122, p < .001). This model explained approximately 15.2% of the variance in feeding choices (Nagelkerke R-squared). The model fit statistics were as follows: Deviance = 953, AIC = 1037, and BIC = 1215.

After holding other variables constant, logistic regression analysis indicated that several factors significantly influenced the likelihood of mothers opting for exclusive artificial feeding rather than exclusive breastfeeding. Mothers aged >35 years were significantly less likely to choose exclusive artificial feeding compared to those aged 25–35 years (AOR = 0.334; 95% CI, 0.160–0.698; p = 0.004). The presence of maternal disease or the use of medications that contraindicate breastfeeding was the strongest barrier identified. These mothers were 12.7 times more likely to choose exclusive formula feeding in the first six months rather than exclusive breastfeeding than those without health-related obstacles (AOR = 12.719; 95% CI 4.102–39.445; p < 0.001). Neonatal weight also played a critical role. Infants born with a weight <2.5 kg had significantly higher odds of being artificially fed than those with a normal birth weight of 2.5–4 kg (AOR = 4.751; 95% CI: 2.075–10.879; p < 0.001).

Moreover, when mixed feeding was compared to exclusive breastfeeding, the primary barriers to EBF included maternal employment (AOR = 3.540; 95% CI: 1.649–7.598; p = 0.001) and maternal disease or medication (AOR = 4.609; 95% CI: 1.506–14.1; p = 0.007).

After testing for potential effect modifiers between the main predictors and demographic variables, no statistically significant interactions were found; therefore, only the main effects of the adjusted predictors were reported.

## Discussion

Despite the known clinical benefits of exclusive breastfeeding, our study found that only 29.9% of infants in northern Jordan received exclusive breastfeeding during the first six months of life. Mixed feeding (formula and breast milk) was the most common feeding practice (46.5%), whereas exclusive artificial feeding was the least common (23.6%). These rates are comparable to previous reports from Jordan. In 2021, the UNICEF reported that only 26% of Jordanian infants were exclusively breastfed in the first six months, which is much lower than the regional average of 34% [16]. A cross-sectional study in southern Jordan similarly reported an EBF rate of 23.95% and a mixed feeding rate of 57.63% [17]. Although our EBF rate is slightly above the national average, it still falls below the WHO goal, which aims for at least half of the infants to be exclusively breastfed by 2025 [18].

Our study also revealed a decrease in breastfeeding duration, with more than half of the infants (58.5%) breastfed for less than 6 months, 21% breastfed for 12 months, and only 20.5% breastfed for more than 12 months. This trend of “early termination” is a recurring theme in the Middle East. A study from western Saudi Arabia showed that although breastfeeding prevalence is high in the first 6 months of life (90%), it decreases to 72% thereafter [19].

This study revealed that the most common reason for introducing artificial milk was to support breastfeeding (63.8%), suggesting that the mothers felt that their milk supply was inadequate to meet the infant’s needs. Insufficient breast milk was also the most commonly reported reason for choosing formula feeding among mothers in a 2017 study in Jordan [20]. Another study in Iran also reported that the primary reason many mothers stopped breastfeeding before their child reached six months or even two years of age was insufficient milk production in the mother’s breasts [21]. A study by Helle et al. in the U.S. also reported that the most common reasons for discontinuing breastfeeding were inconvenience or fatigue (22.6%) and worries about insufficient milk supply (21.6%) [7]. Another study in Poland by Tracz and Gajewska reported that the most commonly cited reasons for discontinuing breastfeeding two months after childbirth were discomfort during nursing, insufficient milk supply, and concerns regarding whether breast milk alone adequately met the baby’s needs [22]. These repeated findings about mothers’ concerns about insufficient milk might be addressed by doctors’ advice to lactating mothers on certain nutritional supplements and practices that have been found to increase milk supply.

No statistically significant association was found between maternal age and infant feeding methods during the first 6 months (p = 0.291). However, we found that mothers aged >35 years were significantly less likely to choose exclusive artificial feeding over EBF compared with those aged 25–35 years. A possible explanation is that older mothers may have greater maternal experience, increased confidence in breastfeeding, or more stable family conditions that support breastfeeding initiation and continuation. In contrast, a study in Australia revealed that mothers under the age of 20 years tended to cease exclusive breastfeeding more quickly in the early postnatal period compared to mothers aged 20–34 years, which was explained by the fact that younger women were more likely to have unintended pregnancies due to a lack of education about contraception [23].

Another study in the U.S. reported that the more educated the mother, the more likely she was to initiate breastfeeding [24]. Furthermore, a study by Tracz and Gajewska in Poland reported a significant correlation between maternal education and the duration of breastfeeding (p = 0.0167); more than half of the participants had a high level of education, and notably, those who breastfed for six months or more tended to have attained higher levels of education [22]. However, in our study, mothers’ educational level was not significantly associated with their mode of nutrition during the first 6 months (p = 0.359). This discrepancy may be attributed to the sociocultural differences between the studied populations.

Regarding maternal employment, while 80.5% of the mothers were unemployed at the time of the study, a critical 19.5% were employed outside the home, with many working long hours. Our study identified a statistically significant association between mothers’ working hours outside the home and their chosen method of nutrition during the first six months (p = 0.019). We discovered that working mothers in our cohort were significantly more likely to use mixed or exclusive artificial feeding. Ryan et al. in the United States (US) found that unemployed mothers were more than twice as likely to breastfeed their infants at six months compared to full-time working mothers, highlighting the challenges that employed mothers face in maintaining breastfeeding [25]. The combination of long hours and insufficient workplace support makes full-time breastfeeding challenging. Therefore, workplace policies should support breastfeeding by allowing adequate breaks for breastfeeding or lactation during work hours. Additionally, flexible working arrangements and extended maternity leave may help working mothers sustain breastfeeding and reduce their reliance on artificial feeding methods.

Interestingly, no statistically significant association was observed between living arrangement (mothers living in a separate household versus with parents or in-laws) and the infant feeding method during the first six months (p = 0.550). While living with parents or in-laws may reduce household financial burdens, this did not seem to affect infant feeding methods in our study population.

In a similar study conducted in Jordan, the authors noted that mothers from low-income families were less familiar with exclusive breastfeeding than their high-income counterparts. This discrepancy can be attributed to the broader access high-income mothers have to various informational sources, both private and public [26]. However, in our study, we found no significant association between monthly family income and the mode of nutrition during the first 6 months (p = 0.067).

Infant characteristics also influence feeding practices. A significant association was found between infants’ birth weight categories and the mode of nutrition during the first 6 months (p = 0.000). Infants weighing less than 2.5 kg at birth were more likely to be artificially fed in the hospital, whereas babies weighing 2.5–4 kg or more than 4 kg were more likely to be breastfed. A study in Greece reported prematurity as a standalone prognostic factor influencing the duration of exclusive breastfeeding and reported that preterm neonates exhibited a higher risk ratio for ceasing breastfeeding earlier than full-term neonates [27]. Prematurity and decreased birth weight can lead to neonatal intensive care unit (NICU) admission, which decreases the chances of breastfeeding. Here, we highlight the importance of doctors’ advice on breastmilk pumping and using it for pre-term babies instead of artificial milk at the hospital, since the NICU serves as more than just a medical treatment facility for newborns; it is a nurturing space designed for both infants and their parents, emphasizing family centered care. Healthcare counseling on breastfeeding also plays a role. Mothers who received prenatal or postnatal breastfeeding counseling were more likely to choose artificial feeding. In contrast, those without counseling tended to choose breastfeeding (p = 0.049), which may reflect gaps in the quality and consistency of breastfeeding support provided by healthcare professionals in Jordan.

Additionally, our findings indicated that mothers who reported an illness or medication that could contraindicate breastfeeding were significantly more likely to opt for formula feeding. Although guidelines emphasize that true contraindications to breastfeeding are very few, mothers often interpret illness or treatment as a signal to stop breastfeeding [28]. Our results align with those of a recent Jordanian study that identified the “presence of a mother’s illness “as a major barrier to breastfeeding [17]. On the other hand, a Saudi study found that mothers who continued breastfeeding despite illness had over five times the odds of continuing EBF compared to those who stopped due to health reasons [29]. Therefore, it is crucial for healthcare providers to inform mothers that most maternal health issues do not necessitate stopping breastfeeding and to educate them about the long-term health benefits of breastfeeding for their infants.

### Limitations

Although we believe our study explored multiple factors affecting the decision to breastfeed in our population, there are several limitations. Firstly, although efforts were made to recruit participants from various healthcare facilities in northern Jordan, the sample may not represent the Jordanian population at large. Similarly, regional variations may exist in infant-feeding practices, and those factors are likely to affect the supply of infants with food, which was not captured in the present study. Secondly, we did not collect data on neonatal intensive care unit (NICU) admission. Preterm and low birth-weight infants are frequently admitted to the NICU immediately after delivery, delaying breastfeeding initiation and increasing early reliance on formula. The lack of NICU information, therefore, represents an unmeasured confounder that may have influenced the observed associations between prematurity, birth weight, and feeding choice. Thirdly, our assessment of infant health is incomplete because it relies on maternal reports to determine outcomes, such as hospital admissions and their causes. A more comprehensive evaluation should include reviews of clinical examinations and patient files, thereby minimizing recall bias and enhancing the precision and depth of findings regarding the infants’ health status.

Future research should eliminate some of the limitations described. For example, more rigorous and representative samples, a longitudinal design, and better assessments of infant health would greatly improve understanding of this important public health issue.

## Conclusion and recommendations

In this study, we sought to identify maternal, infant, and socioeconomic factors influencing infant feeding practices among mothers in northern Jordan. Our findings indicated that the rates of exclusive breastfeeding within the study sample were notably low, with maternal employment, infant birth weight, and maternal illness emerging as the primary determinants of feeding choices. Interestingly, maternal education, family income, and cultural factors such as living arrangements were not statistically significantly associated with feeding choices in this population. These findings underscore the need for healthcare providers to actively promote breastfeeding at all stages of care to increase breastfeeding rates among women in Jordan. Additionally, this study calls on Jordanian policymakers to take action, recognize this issue, and enforce legislation to promote exclusive breastfeeding, such as reducing working hours for lactating mothers and providing spaces for breastfeeding at work or in public places.

## Supporting information

S1 File Anonymized dataset supporting the findings of this study. (XLSX)

S2 File PLOS_human_participants_research_checklist_2025. (PDF)
